# Being Barbie: The Size of One’s Own Body Determines the Perceived Size of the World

**DOI:** 10.1371/journal.pone.0020195

**Published:** 2011-05-25

**Authors:** Björn van der Hoort, Arvid Guterstam, H. Henrik Ehrsson

**Affiliations:** Brain, Body and Self Laboratory, Department of Neuroscience, Karolinska Institutet, Stockholm, Sweden; University of Regensburg, Germany

## Abstract

A classical question in philosophy and psychology is if the sense of one's body influences how one visually perceives the world. Several theoreticians have suggested that our own body serves as a fundamental reference in visual perception of sizes and distances, although compelling experimental evidence for this hypothesis is lacking. In contrast, modern textbooks typically explain the perception of object size and distance by the combination of information from different visual cues. Here, we describe full body illusions in which subjects experience the ownership of a doll's body (80 cm or 30 cm) and a giant's body (400 cm) and use these as tools to demonstrate that the size of one's sensed own body directly influences the perception of object size and distance. These effects were quantified in ten separate experiments with complementary verbal, questionnaire, manual, walking, and physiological measures. When participants experienced the tiny body as their own, they perceived objects to be larger and farther away, and when they experienced the large-body illusion, they perceived objects to be smaller and nearer. Importantly, despite identical retinal input, this “body size effect” was greater when the participants experienced a sense of ownership of the artificial bodies compared to a control condition in which ownership was disrupted. These findings are fundamentally important as they suggest a causal relationship between the representations of body space and external space. Thus, our own body size affects how we perceive the world.

## Introduction

Imagine that during your sleep you shrank to the size of a Barbie doll. Upon awakening, would you feel your body to be small, or would you sense that you were normal in a gigantic world inhabited by giants? This thought experiment illustrates the classical philosophical question of whether one's own body size affects how we perceive the world [1], [2]. But what would happen if we could actually conduct an experiment like this? What if we could achieve a situation in a laboratory setting where people would experience a tiny body or a huge body as their own? How would they then experience the world? In the present study we describe a series of novel experiments that examine this fundamental question.

The perception of object size and distance is traditionally explained as arising from the combination of information from a variety of visual and oculomotor cues [3], [4]. Distance perception is based on binocular disparity [5], [6], oculomotor cues (convergence angle [7] and accommodation [8]), pictorial cues [4] (e.g., occlusion and relative height), and movement cues (motion parallax [9] and depth from motion [10]). Size perception depends on distance cues combined with the retinal size of an object, utilizing the relative size of objects (e.g., the height of a tree is apparent when someone stands next to it), and the principle of size constancy (i.e., a familiar object's size remains constant even when viewed at different distances [3]). However, despite the intuitive idea that the visual system creates a true image of the external world more or less like a video recorder based on retinal and oculomotor information, several theoreticians have suggested that visual perception of objects in the external world partly depends on how one could interact with those objects. According to the ecological approach proposed by Gibson [11], [12], objects in our visual field are perceived in terms of affordances or action possibilities. Importantly, the psychical properties of the observer define the perceived affordances of an object [e.g. 13]–[17]. Going one step further, the embodied cognition movement claims that the possible movement (or effort) one should make to interact with an object directly influences visual perception in a phenomenological manner [e.g. 18]–[20]. In line with this idea are, for example, the findings that the perceived slope of a hill [21] and distances [22] appear larger when participants wear heavy loads. Moreover, objects appear closer when the observer's reachability increases during tool use [23].

Another way to change an observer's repertoire of possible actions is to change the size of the observer's body. A taller person requires fewer steps and less effort to cover a certain distance and therefore should perceive a certain distance to be smaller. To test this hypothesis one should ideally experimentally increase or decrease body sizes within participants. Previous studies have shown that one can experience ownership of a large rubber hand [24], [25] (see Discussion). Others have shown that manipulation of one's apparent hand size has an effect on perceived sizes of objects [26], [27]. Linkenauger et al. (2010) showed that changing the apparent size of one's own hand has a larger effect on perception than a control condition, where another person's apparent hand size was changed. However, since the retinal input of the hand differed between the two conditions, the results found could be attributed to one's unfamiliarity with the size of another person's hand. Thus, previous studies have not conclusively shown an effect of ownership of a body part on visual object size perception in addition to a relative size effect. Moreover, these studies did not test object size or distance perception outside the near-personal space of participants. We do not expect that a change of hand size could change the perception of the entire spatial layout of the environment.

Here, we directly addressed the question of whether the sense of ownership of one's body has an effect on object size and distance perception, in addition to using the body one sees as a relative size cue. We first had to establish that it is possible to experience ownership of artificial bodies of different sizes. We hypothesized that, since earlier studies on limb ownership [28]–[30] and full-body ownership [31]–[35] have emphasized the importance of multisensory integration in body-part-centered reference frames [29], [36], [37], scaling the size of all part-parts symmetrically (up or down) would not affect the body-centered multisensory processes involved and allow ownership of very small or very large bodies. To this end, we used a version of the ‘body swap illusion’ [33] and provided complementary questionnaire and objective physiological evidence that healthy individuals can experience ownership of a very small or very large artificial body (Experiments 1–5, see Table 1).

**Table 1:** Overview of experiments.

Experiment	Description	Measure	Conditions
1	Small body illusion	Questionnaire	Sync, Async
2	Large body illusion	Questionnaire	Sync, Async
3	Small body illusion	SCR	Sync, Async
4	Large body illusion	SCR	Sync, Async
5	Barbie doll illusion	Questionnaire	Sync
6	Size perception	Verbal size estimation + Questionnaire	Small-Sync, Normal-Sync, Large-Sync
7	Size perception	Hand aperture + Questionnaire	Small-Sync, Normal-Sync, Large-Sync
8	Size perception	Hand aperture	Small-Sync, Small-Async, Large-Sync, Large-Async
9	Distance perception	Verbal distance estimation	Small-Sync, Small-Async, Normal-Sync, Large-Sync, Large-Async
10	Distance perception	Walking distance	Small-Sync, Small-Async, Large-Sync, Large-Async

We then carried out a series of experiments to investigate whether the size of the owned body influenced the perceived size and distance of objects presented in front of the participants (Experiments 6–10, see Table 1). Importantly, in these experiments, participants received identical visual information in the illusion condition and a control condition, i.e. only the sense of ownership differed between otherwise equivalent conditions. Moreover, we examined the effect of own body size on visual perception both within and beyond personal space. We hypothesized that owning an extremely large body (400 cm) renders objects to be judged as smaller and closer by, and vice versa for owning an extremely small body (80 cm or 30 cm), and that these effects are larger in the ownership condition. Furthermore, we expected this effect to be present both at small and large distances. Our results provide compelling support for the idea that the size of one's own body directly influences the perception of the size of the entire external world.

## Results

### Owning different sized artificial bodies

**Figure 1:**
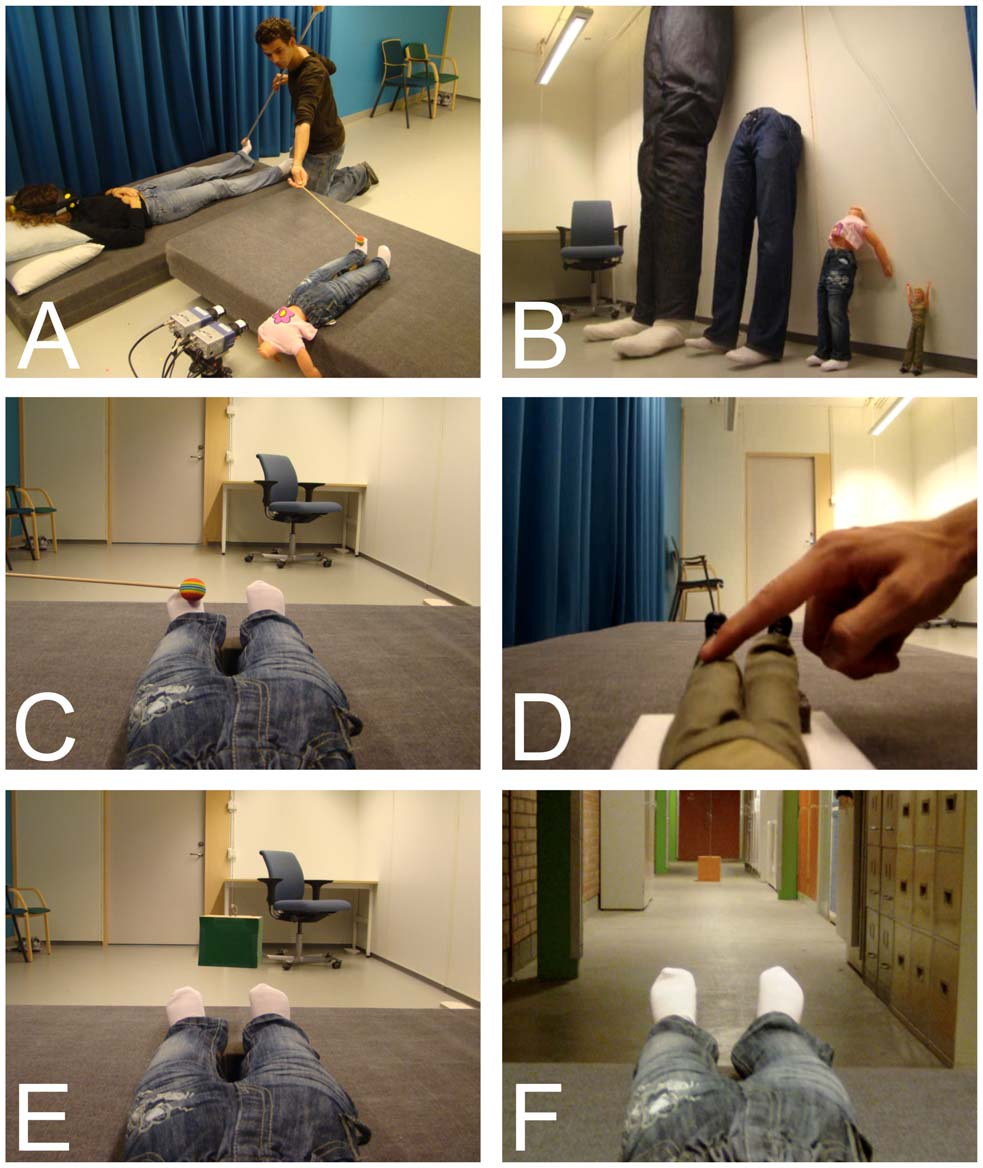
Experimental set-up. This figure displays the main experimental set-up (A), the four artificial bodies (B), and the image seen by participants during visuo-tactile stimulation (C), the Barbie doll experiment (D), object size estimation (E), and distance estimation (F).

The first goal was to test the hypothesis that robust perceptual illusions of owning tiny or huge bodies could be induced. To this end, we used a version of a previously published full-body illusion in which participants experience a mannequin's body as their own [33]. In the current experiments, participants lie on a bed while wearing a set of head-mounted displays (HMDs). The HMDs are connected to a pair of cameras mounted on a tripod placed behind and facing an artificial body lying on a bed next to the participant (see Figure 1A,B). This setup allows participants to see a real-time 3D image of an artificial body from the first-person perspective, as though their head were tilting forward and they were looking directly at the doll (see Figure 1C). The experimenter then synchronously touches the participant's body (out of view) and the artificial body (in view) with a small rod. This stimulation creates the illusion that the artificial body is the participant's own body and that it senses the touch of the rod. For a normal-sized artificial body, this happens as a consequence of the brain's attempt to reconcile the spatially and temporally correlated visual and somatic signals, resulting in the multisensory perception that the touched plastic body is one's own [33]. In Experiments 1–5, we demonstrate that this illusion can be induced using a small (80 cm or 30 cm) doll or an enormous 400 cm artificial body.

**Figure 2:**
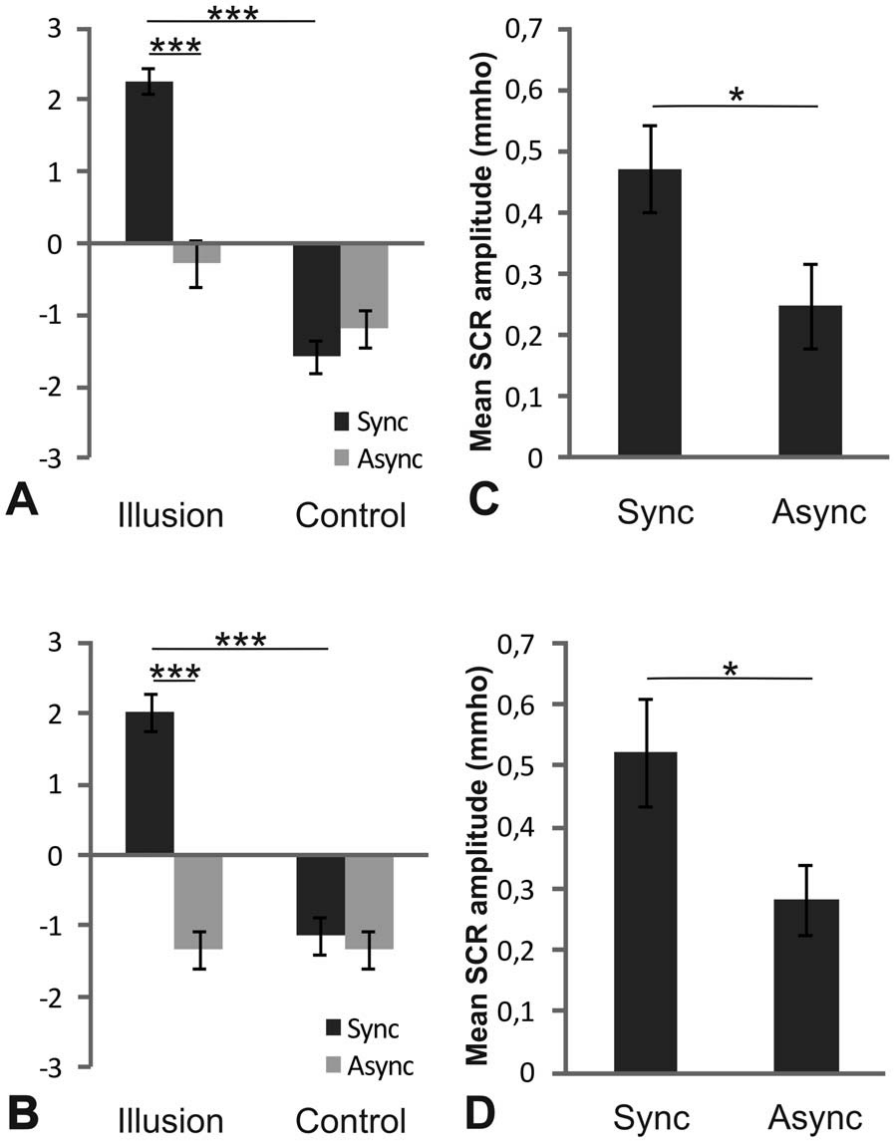
Results of Experiments 1–4: Illusory ownership of tiny and huge artificial bodies. Average scores on illusion statements and control statements (see Table S1) after synchronous and asynchronous touching of small body (A) and large body (B), and average threat-evoked SCR after a period of synchronous and asynchronous touching of the small body (C) and the large body (D). * p<0.05, *** p<0.001. Error bars indicate SEM.

In the experimental condition, the participant's body and the artificial body were touched synchronously for four minutes; in the control condition, the two bodies were touched asynchronously for the same duration, as this mode of stimulation is known to diminish the body-swap illusion significantly [33]. In Experiments 1 and 2, participants were asked to report their experiences by completing a questionnaire after each of these two conditions. The questionnaire consisted of three illusion statements designed to capture the subjective feeling of ownership of the artificial body and four statements to control for the effects of suggestibility and task compliance (see Table S1). The results showed that the participants strongly affirmed the illusion and gave significantly higher scores to the illusion statements compared with the control statements, but only during the synchronous condition (significant interaction between statement type and condition for the small body: n = 15, F(1, 14)  = 21.059, p<0.001, and the large body: n = 14, F(1, 13)  = 69.394 p<0.001; repeated measures ANOVA, see Figure 2A,B) (see Text S1 and Figure S1 for results for each individual statement).

Objective evidence for these illusions was obtained in Experiments 3 and 4. Here, we measured the skin-conductance response (SCR) evoked by physically harming the artificial bodies, representing a physiological index of the illusion [33]. After a period of experiencing the illusion with synchronous visuotactile stimulation, or the asynchronous control condition, the participants observed a knife cutting the lower abdomen of the artificial body and their SCR was registered. The threat-evoked SCR was significantly higher after the synchronous condition than after the asynchronous condition (small body: n = 18, Z = −1.851, p<0.05; and large body: n = 16, Z = −2.223, p<0.05; Wilcoxon signed-rank tests) (see Figure 2C,D). Thus, the participants responded emotionally as if the small and large artificial bodies were their own. This, in combination with the questionnaire data from the two first experiments, shows that the body-swap illusion works on very small or extremely large artificial bodies.

**Figure 3:**
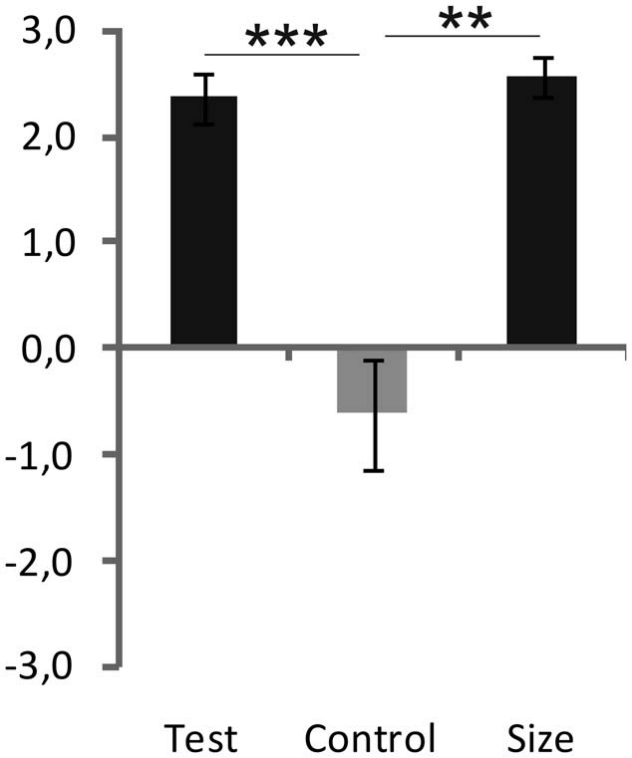
Results of Experiment 5: Illusory ownership of the body of a Barbie doll. Average scores for illusion statements, control statements and statements regarding the size of seen objects (see Table S1). ** p<0.01, *** p<0.001. Error bars indicate SEM.

In a final demonstration of this illusion, we wanted to investigate the subjective feeling of body ownership with a tiny Barbie doll (30 cm) (Experiment 5, see Figure 1D). The motivation for this last experiment was two-fold. First, we wanted to demonstrate that the small-body illusion works with an extraordinarily small body (a Barbie doll). Second, we wanted to show that the sight of people and well-known objects would not break the illusion. The basic method of this experiment was similar to the method of Experiments 1 and 2. The doll's body was subsequently touched with a small rod, a pencil, and the experimenter's finger. After the four minutes of synchronous visuotactile stimulation, participants gave significantly higher ratings on the illusion statements compared to the control statements (t8 = 6.037, p<0.001; paired t-test, see Figure 3). In the same questionnaire (see Table S1), participants also agreed on the illusion statements regarding the size of the pencil and the finger they had seen (t8 = 4.599, p<0.01; paired t-test, see Figure 3) (see Figure S2 for results for each individual statement).

Both the finger and the pencil appeared to be gigantic to the participants, despite their high familiarity with these items. Thus, even the sight of familiar objects and people fails to dispel the body-swap illusion and its effect on visual perception. Anecdotally, most participants were not aware of the extremely small size of the doll that they felt ownership of. Instead, they experienced themselves to be located in a “giant world”.

### The effect of body size on visual perception

Next, we turned to the main goal of the study, which was to employ these illusions to test the hypothesized causal relationship between one's body size and the perceived distances and sizes of external objects. First, we examined the effect of own body size on object size perception. We showed cubes of different sizes at a constant distance from the cameras after the induction of the body-swap illusions with the various bodies (small, 80 cm; normal, 180 cm; and large, 400 cm). Importantly, the height of the cameras, the distance between the cameras, and the distance between camera and target object remained identical across all trials in these experiments. Therefore, other size and distance cues, such as retinal image, binocular disparity, accommodation, and eye convergence, remained constant across all trials. The only factors that varied were the artificial bodies seen from a first person perspective (Experiments 6–8) and the sense of owning those artificial bodies (Experiment 8). We also ensured that the target objects had different colors in each trial to prevent memory from confounding the results. The participants had to report the size of the cubes either verbally (Experiment 6) or manually (Experiments 7 and 8) (see Figure 1E).

**Figure 4:**
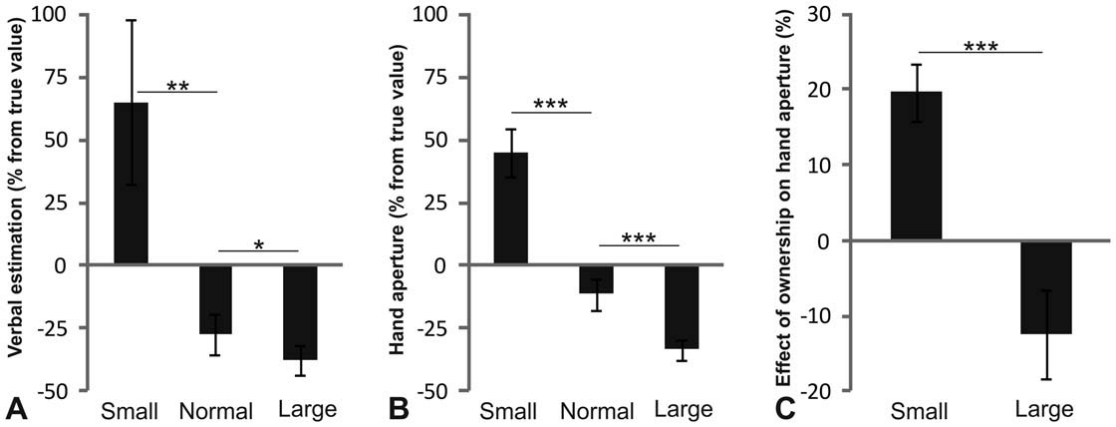
Results of Experiments 6–8: Own body size effect on size perception. The body size effect on verbal size estimation (A) and hand aperture (B) as a percentage deviation from the average estimation of all trials and the effect of the ownership illusion on hand aperture as a percentage deviation from corresponding asynchronous condition (C). * p<0.05, ** p<0.01, *** p<0.001. Error bars indicate SEM.

Compared with the illusion of owning a normal-sized artificial body, participants gave significantly higher verbal estimates of cube size during the small-body illusion (n = 14, Z = 2.982, p<0.005; Wilcoxon signed-rank test) and significantly lower estimates when experiencing the large-body illusion (n = 14, Z = −1.713, p<0.05; Wilcoxon signed-rank test) (Experiment 6, see Figure 4A). Moreover, when participants were requested to report the size of the target objects by holding up their hands and representing the width of the cubes as the distance between their hands (Experiment 7), we obtained the same results. Compared with the normal body, bimanual object-size estimations were significantly higher during the small-body illusion (n = 14, Z = 3.296, p<0.001; Wilcoxon signed-rank test) and significantly lower during the large-body illusion (n = 14, Z = −3.296, p<0.001; Wilcoxon signed-rank test) (see Figure 4B). Importantly, the strength of the illusion of body ownership did not differ for the different artificial body sizes during experiments 6 and 7 (see Text S1 and Figure S3).

Next, we went on to demonstrate that the very sense of owning the body contributed to these effects. To this end, we manipulated the strength of the illusion by applying the synchronous and asynchronous modes of visuotactile stimulation and again measured the object size perception using the bimanual response measure because this was the most exact (Experiment 8). Importantly, the overestimation of object sizes after experiencing the small-body illusion and the underestimation of these during the large body-illusion were significantly more pronounced after a period of synchronous touches than after a period of asynchronous touches (interaction effect of timing × body size; n = 20, F(1,19)  = 17.789, p<0.001; repeated measures ANOVA) (see Figure 4C). Thus, the effect of body size on object size perception was greater when the participants sensed ownership of the artificial body. This excludes the possibility that the effect found in Experiments 6 and 7 was driven solely by using the body in the image as a relative size cue.

**Figure 5:**
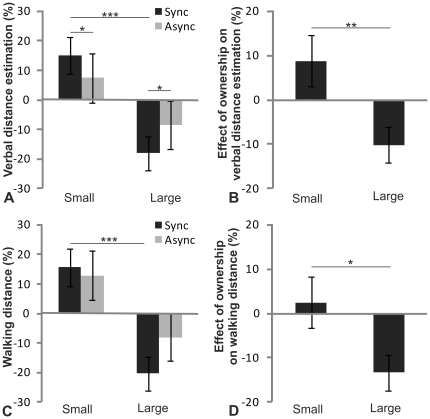
Results of Experiments 9 and 10: Own body size effect on distance perception. The body-size effect on verbal distance estimation as a percentage deviation from average estimations (A) and as percentage deviation from the corresponding asynchronous condition (B). The body-size effect on walking distance as a percentage deviation from average estimations (C) and as a percentage deviation from the corresponding asynchronous condition (D). * p<0.05, ** p<0.01, *** p<0.001. Error bars indicate SEM.

Next, we investigated whether the changes in illusory own body size also produced changes in the perceived distance of external objects. In Experiments 9 and 10, we addressed this question using explicit and implicit measures of distance perception, respectively. Again, the image seen through the HMDs was identical for all conditions except the size of the artificial body. Therefore, changes in distance perception can only be subscribed to the size of the artificial bodies and the illusionary ownership of those bodies and not to other cues. In each trial, the participants viewed one of the three artificial bodies (small, normal, or large) lying on a bed in different hallways and corridors. After a period of experiencing the illusion or the asynchronous control stimulation, the participants saw objects at various distances from the camera (4 m, 8 m, and 16 m) and verbally reported the perceived distances (Experiment 9, see Figure 1F). As compared with owning the normal-sized body, participants estimated object distances to be significantly larger when owning the small body (n = 25, Z = 2.069, p<0.05; Wilcoxon signed-rank test), and significantly smaller when owning the large body (n = 25, Z = −3.109, p<0.005; difference between small body and large body: n = 25, Z = 3.872, p<0.001; Wilcoxon signed-rank test) (see Figure 5A). Importantly, this effect of body size on distance perception was greater in the illusion condition than in the control condition (small body: n = 25, Z = 1.978, p<0.05; large body: n = 25, Z = −1.901, p<0.05, see Figure 5A; difference between small body and large body: n = 25, Z = 2.300, p<0.05, Wilcoxon signed-rank tests, see Figure 5B).

In Experiment 10, we replicated this finding using an implicit behavioral measure. After the stimulation periods, the participants were asked to stand up and walk with their eyes closed towards the point where they perceived the object to be located. The participants walked a longer distance during the small-body illusion than in the large-body illusion (n = 28, Z = 4.600, p<0.001, see Figure 5C). Again, this body-size effect was stronger in the illusion condition than in the control condition (n = 28, Z = 2.289, P<0.05, Wilcoxon signed-rank test; see Figure 5D). This finding, taken together with the results from Experiments 6–9, provides very strong evidence that the size of the body we experience ownership of has a direct effect on the perception of object size and distance.

## Discussion

We found two main results. First, we induced full body illusions where participants experienced ownership of abnormally large and small artificial bodies. We theorize that the size of the artificial body used in this type of illusion is potentially unlimited as long as all the parts of the artificial body are scaled (up or down) proportionally. This might explain why the present illusion works well with both large and small bodies, whereas earlier studies on illusionary ownership of small and large rubber hands have found asymmetrical results, with larger hands producing greater effects [24], [25]. Unlike the present illusions, altering the size of one body part disproportionally could be interpreted as a change in distance with respect to the head and eyes. The illusion of owning a large hand can be explained by an illusion of a decreased distance between the hand and the eyes of the observer, but the reverse pattern is harder to obtain since an increase of the distance between the hand and the eye would be accompanied by an elongated neck or arm which would imply an incongruent body representation.

Second, we found that the very sense of owning a different sized artificial body results in a change in the perception of sizes and distances in the external world. This finding provides support for the embodied cognition movement and provides powerful evidence in favor of the idea that the body provides a metric for space perception (see introduction). However, our results go beyond earlier studies for three reasons. Firstly, the body size effect on space perception extends beyond near-personal space (at least up to 12 m), whereas former studies have only described such an effect near the hand. Our results suggest that one's own body size serves as an approximate reference for the entire external world in view and not just within one's personal space. Secondly, we found similar results on implicit and explicit measures of perception within the same experimental paradigm. Thirdly, we are the first to explicitly describe the additional effect of body ownership beyond merely using the body (part) in sight as a relative size cue. Importantly, the retinal input was identical during the synchronous (ownership) and asynchronous (no ownership) conditions, thus the stronger body size effect on visual perception during the synchronous condition can only be explained by the presence of body ownership.

Psychologically, our results could be explained by at least two not mutually exclusive mechanisms. First, the world might appear smaller to a larger observer because the effort to interact with that environment decreases, and vice versa [18]–[20]. Second, because the representation of allocentric space is considered to be functionally linked to ego-centric representations [38], [39], scaling the latter (intrapersonal and near personal space) could produce changes in the representations of far extrapersonal space. Thus, when scaling the size of the entire body proportionally as in the present full-body illusions, the effects on space perception could become ‘global’ causing changes in all representations of space (body-part-centered, ego-centric and allocentric), rather than just affecting space near a single limb, as occurs when the size of a single limb is manipulated using the rubber hand illusion [24], [25]. Our results suggest that these interactions are causal because changing the size of the body for the same participant during an experimental session changed perception of space far from the body in a systematic manner.

What could be the neuronal mechanism for the basic interaction between body representation and space perception? The body-swap illusion itself is likely to involve the integration of visual, tactile, and proprioceptive information in egocentric reference frames in multisensory areas in the frontal and parietal lobes [29], [33], [37]. The interaction between the multisensory representation of the body and the processing of visual signals is probably mediated by feedback projections from the frontoparietal multisensory areas to higher-order visual areas in the occipital, posterior parietal, and temporal lobes [40]–[44]. These projections are likely to target areas in both the ventral and dorsal streams, as we observed the body-size effect both by an explicit measure (verbal report) and implicit measures (hand aperture and walking) [45], [46]. It is even possible that such modulatory effects could be present as early in the sensory process as the primary visual cortex [47]. The possibility that the posterior parietal cortex is involved in mediating the present perceptual effects is consistent with the neurological observation that migraine or focal epilepsy centered over the parietal lobe can produce the ‘Alice in Wonderland syndrome’ where people sometimes experience their entire body to be expanding or shrinking in size with accompanying changes in the perceived size of external objects and people [48]–[50].

The present findings could have important clinical and industrial applications in tele-robotics and virtual-reality research, in which an interesting new direction is to project the feeling of ownership onto advanced humanoid robotic devices and simulated bodies [51]–[53]. The present results provide the proof of concept that this could work with very small or very large humanoid robots. For example, a surgeon could experience a full-body illusion of “being” a microrobot performing surgery inside the patient's body or an engineer could perceive ownership of a gigantic humanoid robot repairing deep-sea oil-drilling devices.

In conclusion, the size of the body we own plays an important role in how we visually perceive our surroundings; the world appears larger to a small observer and smaller to a large observer. These results contribute to resolving a centuries-old debate in philosophy and psychology and demonstrate that the visual perception of object size and distance directly depends on the multisensory body representation. Thus, the sense of one's own body affects how we visually experience the world.

## Materials and Methods

### Ethics Statement

All participants gave their written informed consent prior to participating in the experiment. The participants in this manuscript have given written informed consent to the publication of their image in this manuscript. All experiments were approved by the Regional Ethical Review Board of Stockholm.

### Participants

We recruited a total of 198 naive, healthy adult participants for the 10 experiments, with the following numbers of volunteers for each experiment: Experiment 1: 15 (6 females, 27.6 years (mean age)±1.6 years (SE)); Experiment 2: 14 (4 females, 28.1±2.4 years); Experiment 3: 30 (17 females, 24.4±0.6 years); Experiment 4: 25 (9 females, 28.4±1.5 years); Experiment 5: 9 (2 females, 29.8±1.9 years); Experiment 6: 16 (9 females, 26.1±1.5 years); Experiment 7: 16 (8 females, 30.1±3.3 years); Experiment 8: 20 (10 females, 31.4±2.9 years); Experiment 9: 25 (9 females, 28.4±1.5 years); Experiment 10: 28 (16 females, 26.4±1.2 years).

We chose to recruit naive participants for all experiments in order to prevent participants from adjusting their response to the ones they gave on previous experiments (e.g. explicit versus implicit). The use of such a serial design with many individual experiments instead of a factorial design, where a large number of conditions and tests would have to be administered to the same participants, promises to give more reliable results.

### Video technology and artificial bodies

The participants wore a set of head-mounted displays (HMDs) (Cybermind Visette Pro PAL, Cybermind Interactive, Maastricht, the Netherlands; display resolution = 640 × 480, field of view = 71.5°) that were connected to two synchronized color CCTV cameras (Protos IV, Vista, Wokingham, Berkshire, United Kingdom). The distance between the cameras (9 cm) was fixed for all participants. The image was directly transmitted to the HMDs without any software conversion, so there was no noticeable delay.

The participants initially saw a homogenous gray screen because the cameras connected to the HMDs were covered by a gray cloth. When the experimental trial began, the cloth was removed and the participants saw a real-time 3D image of an artificial body (Experiments 1–4, 6–8). This was achieved by placing various artificial bodies in front of the cameras (see below). In Experiments 5, 9, and 10, we used prerecorded 3D images because the very small size of the Barbie doll made the application of the touches in real time difficult (Experiment 5), and because we needed to present different scenes for different trials (Experiments 9 and 10). Thus, in these latter experiments, we used a different set of HMDs (Cybermind Visette 45, Cybermind Interactive, Maastricht, the Netherlands; display resolution 1280 × 1024, 45° field of view), which allowed the presentation of prerecorded digital images in 3D. These recordings were made with two Sony HD Camcorders (Sony Electronics, San Diego, CA, USA; resolution = 1280 × 1024), and the image files from the two cameras were synchronized with custom software (written by Dr. Alexander Skoglund).

Four different artificial bodies were used in the study: a life-sized 180 cm mannequin with the lower 100 cm visible to the participants in the HMDs, an enormous artificial body (of 400 cm, with 220 cm visible) made of wood, a small doll (80 cm, 45 cm visible), and a tiny doll (30 cm, 17 cm visible) (Figure 1B). With the exception of the Barbie doll used in Experiment 5, all artificial bodies wore custom-made clothes (a white t-shirt and jeans) to match the appearance across bodies. During all experiments, participants could see the legs and lower abdomen of the artificial body from a first-person perspective (see Figure 1C–F). Participants could also see a part of the testing room including a door, a desk, and a chair (Experiments 1–7 and 10) or a hallway/corridor (Experiments 8 and 9).

### Visuotactile stimulation

We used two visuotactile stimulation conditions. In the synchronous condition, we touched the participant's body and the artificial body simultaneously and at corresponding locations with a small ball attached to a rod. In the asynchronous condition, we applied the touches to the participant's body and the artificial body in an alternating manner, stimulating different parts of the two bodies. Only the synchronous condition elicited a vivid body illusion, thus allowing us to compare otherwise identical conditions. In Experiments 1–4 and 8–10, we tested both the synchronous and asynchronous conditions; in Experiments 5–7, we only included the synchronous one. The tactile stimuli were strokes applied along the length of the right and left lower leg and the left foot (each stroke was approximately 30 cm (legs) and 10 cm (foot) long and lasted approximately one second). The size of the ball that touched the artificial body was proportional to the size of that artificial body (varied from 3 cm to 10 cm in diameter) to maintain a match between the visual impression of this object and the tactile sensations of the ball (which always had a diameter of 6 cm) touching the person's real leg. To further match the visual and tactile stimuli, the length of the strokes applied to the artificial bodies was kept proportional to the artificial body's size. For example, strokes applied to the large artificial body were about twice as long as strokes applied to the participant, ensuring a match between what the participants saw and felt relative to the body size. In both conditions, approximately 20 such visuotactile stimuli were applied per minute.

### Procedures

#### Experiments 1 and 2

Participants underwent two sessions of visuotactile stimulation, each session lasting four minutes. One session corresponded to the synchronous mode of stimulation and the other to asynchronous stimulation. After each session, the participants completed a questionnaire. Participants rated their agreement on seven statements on seven-point Likert scales. Three test statements (T1–3) were designed to capture the illusory feeling of owning an artificial body, and four control statements (C1–4) were designed to control for task compliance and suggestibility (Table S1).

#### Experiments 3 and 4

The skin conductance evoked by threatening the body was registered after six sessions, each lasting 90 seconds. Three sessions corresponded to the synchronous condition and the other three to the asynchronous condition. The order of these sessions was counterbalanced across participants. At the end of each session, we used a knife to cut the lower abdomen of the artificial body and we registered the threat-evoked skin conductance responses. The knife was seen moving toward the right side of the artificial body's abdomen and then cut the abdomen from right to left. This whole event took approximately 3.5 seconds, during which the knife was in contact with the abdomen for about 2 seconds. We recorded the skin conductance of participants with a Biopac system MP150 (Goleta, USA) (parameters: gain switch = 5 mmho/V; CAL2 Scale Value = 5). An electrode was attached to the participant's right-hand index finger and another to the right-hand middle finger. We used Signa electrode gel (Parker Laboratories, Inc., Fairfield, USA) to improve the signal-to-noise ratio. The data was registered at 100 samples per second and was processed with the Biopac software package (Acknowledge for Windows ACK100W). During the data acquisition, we pressed a key to indicate the timing of each knife cut in the raw data file. The skin-conductance response (SCR) was identified as the peak amplitude within 5 seconds after the onset of each knife threat. The amplitude was calculated by subtracting the minimal conductance value that preceded the maximal conductance value. For each participant, we calculated the average amplitude in the synchronous condition and in the asynchronous condition but only when an SCR could be distinguished in at least 50% of the knife threats. Participants who responded to fewer than 50% of the knife threats were excluded from further analysis. In Experiments 1–4, the participants were not blindfolded before entering the testing room in accordance with the previously published protocols [33].

#### Experiment 5

To induce the illusion, participants experienced a total of four minutes of synchronous visuotactile stimulation on the legs of their real body and on the legs of a Barbie doll seen through the HMDs. In contrast to Experiments 1 and 2, participants saw a prerecorded video of the Barbie doll's body from a first person perspective because the small size of the doll rendered real time synchronous stimulation impractical. Importantly, the object that we used for touching the doll changed during the experiment. We started with an unfamiliar rod for two minutes, which was followed by a familiar object (a pencil) during the third minute, and we ended each session with the experimenter's finger directly touching the doll for one minute (Figure 1D). To match the visual impressions of these three different stimuli touching the doll's legs and feet, we used a very large rod (to match the small rod and pencil) and the experimenter's whole hand (to match the finger) to touch the participant's real legs and feet. To synchronize the touches as well as possible, the experimenter listened to an audio file that was made during the recording of the video, providing instructions on the type, location, and timing of touching. After four minutes of such stimulation with rods, pencils, and fingers, the participants completed a questionnaire very similar to those used in Experiments 1 and 2 with the addition of questions about their estimates of the sizes of the experimenter's hand and the pencil (Table S1).

#### Experiment 6

Participants were blindfolded prior to the experiment to prevent them from seeing the test objects and artificial bodies. Object size perception was measured in nine trials, with three trials for each of the three body-size conditions (small, normal, and large). Each trial consisted of 90 seconds of synchronous visuotactile stimulation as described above, followed by the visual presentation of a cube (hanging on a fishing line) entering the field of view from above (Figure 1E). The cube remained visible above the artificial body for 2 seconds (the experimenter was never visible). The participants were then asked to estimate and verbally report the size of the cube using half-centimeter accuracy (e.g., 20 cm or 20.5 cm). We used different sized cubes for each of the three trials per condition; the sizes of the cubes were 10 cm, 20 cm, and 40 cm. These cubes were also of different colors in all nine trials, preventing participants from recognizing cubes across body-size conditions. We further randomized the order of the conditions and the order of the presentation of the different cubes. When we changed the artificial body, we covered the camera with a piece of grey cloth to prevent participants from seeing the experimenter.

When all nine trials had been completed, the participants were again blindfolded and guided to a table where they could not see the artificial bodies, the test objects, or the setup. Here, they completed a questionnaire about their experiences in relation to the body illusion (Table S2). The questionnaire contained two illusion statements and one control statement for each of the three bodies.

#### Experiment 7

The procedures and rationale were the same as for experiment 6, but instead of verbally reporting the size of objects the participants indicated size estimations by using their hands. Thus, after the cube had disappeared from view the participants were instructed to quickly raise their hands and to hold them straight up above their head (out of sight). They were asked to indicate the size of the cube as the width between the palms, and to maintain their hands in this position for 10 seconds while the experimenter measured this distance with a ruler. As in experiment 6, the participants completed the short questionnaire at the end of the experiment rating the strength of the illusory experiences.

#### Experiment 8

Here, the goal was to demonstrate a direct link between the illusion of owning the artificial bodies and changes in object size perception. Thus, we used the same bimanual estimation procedure as in experiment 7 but compared the synchronous condition to the asynchronous condition serving as a control. We only used the small and the large artificial bodies in this experiment, resulting in a 2 × 2 factorial design (timing × body size). Thus, this experiment consisted of four conditions, each repeated three times for a total of twelve trials. The order of the four conditions was randomized among participants. We predicted a significant interaction between the two factors (timing and body) in the factorial design.

#### Experiment 9

Here, we tested the hypothesis that a larger perceived body size would result in objects appearing closer and vice versa. To prevent participants from seeing the corridors that we used for the prerecorded movies, we blindfolded them before they entered the testing facilities. In the testing room, the participants lay on the bed and wore HMDs as in Experiments 1–4 and 6–8. We used five conditions: asynchronous touching with the small (1) and large bodies (2) and synchronous touching with the small (3), normal (4), and large (5) bodies. Each condition was repeated three times in three separate trials. The experimenter could synchronize the touches he applied to the participant with the touches the participant saw in the HMDs by listening to an audio file, which informed the experimenter about the place and timing of the touches via a set of earphones. After 80 seconds of synchronous or asynchronous visuotactile stimulation, the HMD screen went black for two seconds. The participants then saw a cube placed on the floor of the corridor at some distance in front of the artificial body, which was still in view (Figure 1F). These cubes were presented at distances of 4 m, 8 m, or 16 m from the cameras. The participants were then asked to estimate and verbally report the distance between the cube and their “head,” and they were instructed to do so within eight seconds. For each trial, we presented a different prerecorded scene showing a corridor or hallway that the participant had not seen before to prevent any memory strategies from biasing the results. Furthermore, across individuals we matched the presentation of the three different artificial bodies in the six different scenes. The orders of bodies, distances, scenes, and conditions were also randomized across individuals.

#### Experiment 10

We used the same experimental procedures as in experiment 9, but now the participants indicated the distance to the objects in the corridor by actually walking toward them. After each trial of visuotactile stimulation and presentation of the target cube, the screen went blank and the participants were instructed to stand up and walk with their eyes closed to the point in the corridor in front of them where they perceived the object to be located. We used the distance walked as the behavioral measure. In this experiment, we only tested the large-body and small-body conditions, and only used one target distance (8 m). This allowed us to reduce the number of trials (six in total; three of each condition) because the walking procedure took much longer than the verbal reports. This also allowed us to adopt a 2×2 factorial design, and here, we predicted an interaction between the factors of timing (synchronous or asynchronous) and body size (small or large).

### Statistical analysis

For Experiments 6–9 we normalized the data points to a standard size/distance prior to averaging within participants. This procedure is valid as we noted that the effects on object size and distance perception (see Results) were qualitatively similar for the different object sizes and distances tested (data not shown). We then tested whether the averaged data fitted the requirements for normal distributions using a Shapiro-Wilk test. For normally distributed data sets, we used t-tests to analyze the differences between two conditions, and repeated-measures ANOVAs to test for interaction effects. For data sets that were not normally distributed we used nonparametric Wilcoxon signed-rank tests to analyze differences between two conditions. We used one-tailed tests because we had strong a priori expectations in all Experiments. The alpha value was set to 0.05 for all statistical tests.

## Supporting Information

Figure S1 **Questionnaire results for the small and large body illusion.** Results displayed for the small body (A) and large body (B) questionnaire experiments displayed for each individual statement (See Table S1). T1–T3: test statements 1–3. C1–C4: control statements 1–4, * p<0.05, *** p<0.001. Error bars indicate SEM.
(TIF)

Figure S2 **Questionnaire results for the Barbie doll illusion.** Results are displayed as an average per individual statement (A), and per statement type (B) (See Table S1). T1–T3: test statements 1–3, C1–4: control statements 1–4, S1–2: size statements 1–2, ** p<0.01, *** p<0.001. Error bars indicate SEM.
(TIF)

Figure S3 **Combined questionnaire results for size perception in Experiments 6 and 7.** Results displayed according to statement type (illusion and control) and body size (A), and the illusion strength (defined as average score for illusion statements minus average score on control statement for different body sizes) (B). *** p<0.001, n.s.  = difference is not significant. Error bars indicate SEM.
(TIF)

Table S1 (DOCX)

Table S2 (DOCX)

Text S1 (DOCX)
