# Supplementary figures and images for: Being Barbie: The Size of One’s Own Body Determines the Perceived Size of the World
Source: PLoS One. 2011 May 25;6(5):e20195. doi: 10.1371/journal.pone.0020195 (PMC3102093; doi:10.1371/journal.pone.0020195)

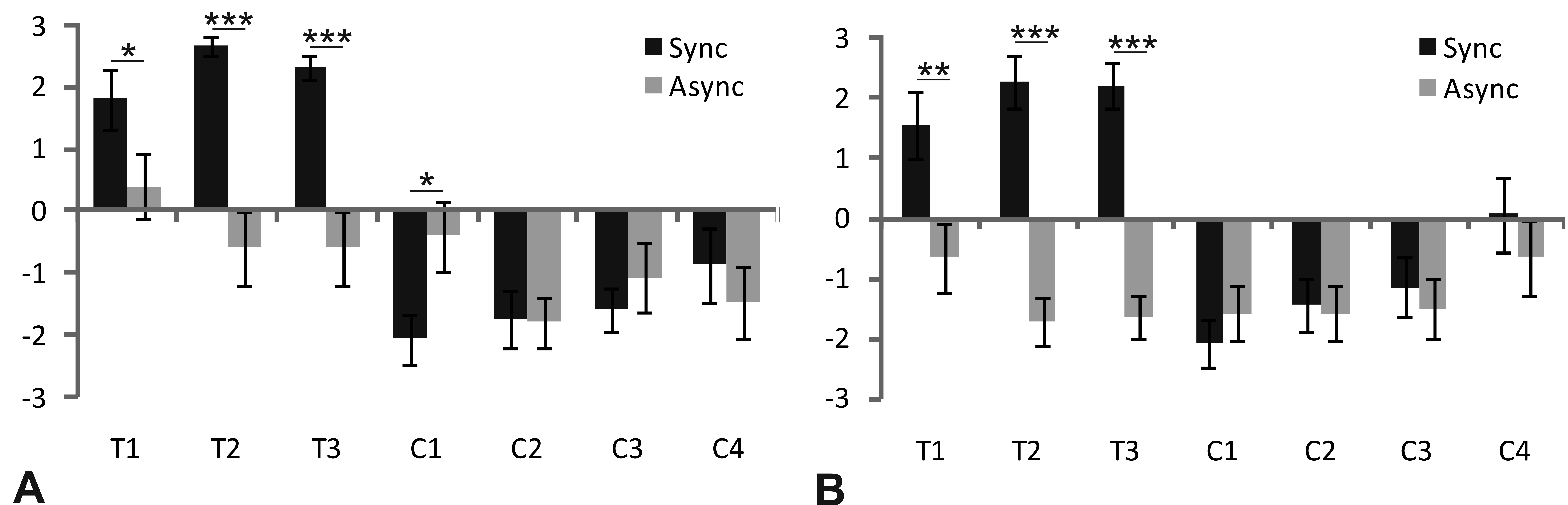

Supplement: Figure S1 — Questionnaire results for the small and large body illusion. Results displayed for the small body (A) and large body (B) questionnaire experiments displayed for each individual statement (See Table S1). T1–T3: test statements 1–3. C1–C4: control statements 1–4, * p<0.05, *** p<0.001. Error bars indicate SEM. (TIF) [file pone.0020195.s001.tif]

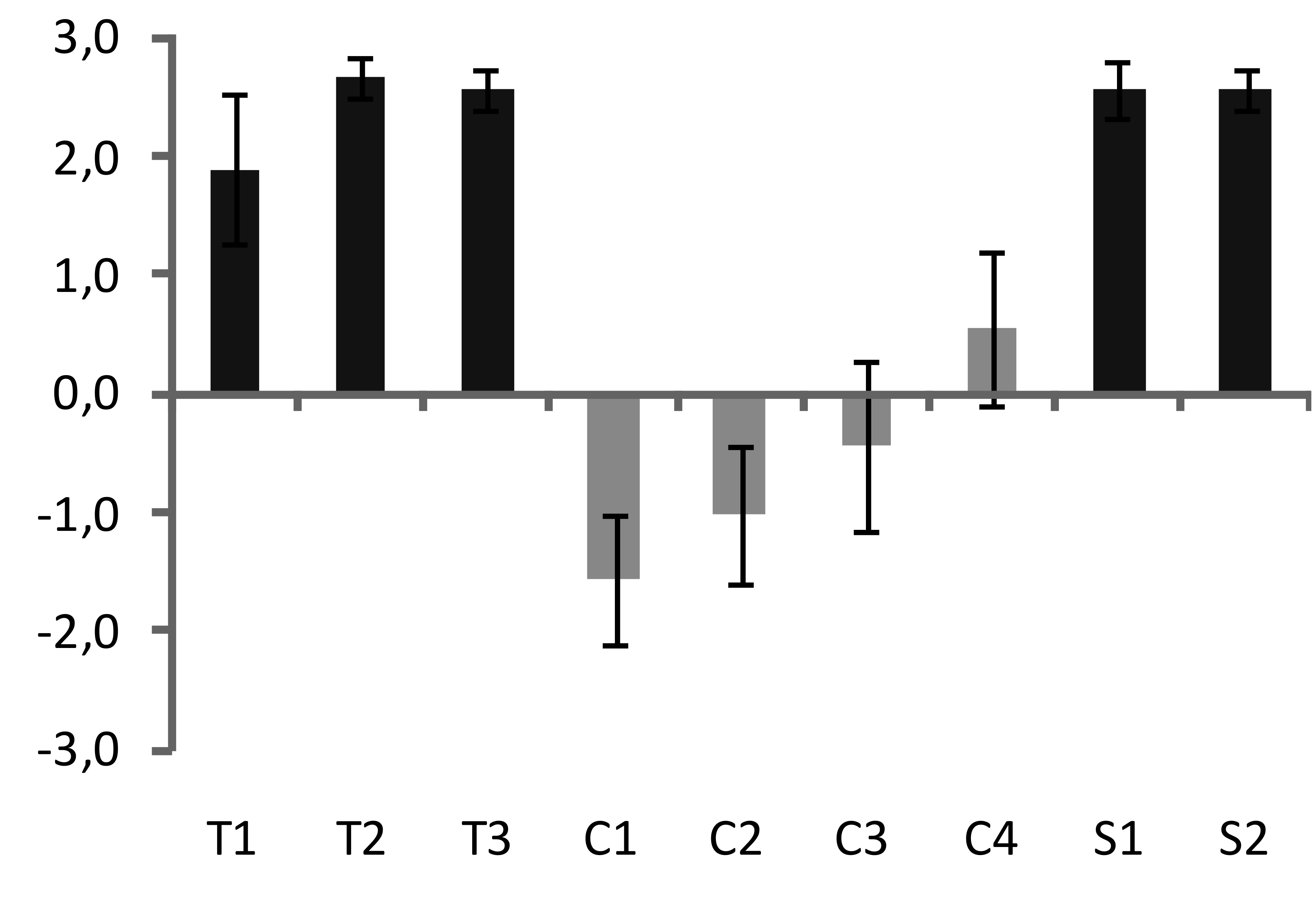

Supplement: Figure S2 — Questionnaire results for the Barbie doll illusion. Results are displayed as an average per individual statement (A), and per statement type (B) (See Table S1). T1–T3: test statements 1–3, C1–4: control statements 1–4, S1–2: size statements 1–2, ** p<0.01, *** p<0.001. Error bars indicate SEM. (TIF) [file pone.0020195.s002.tif]

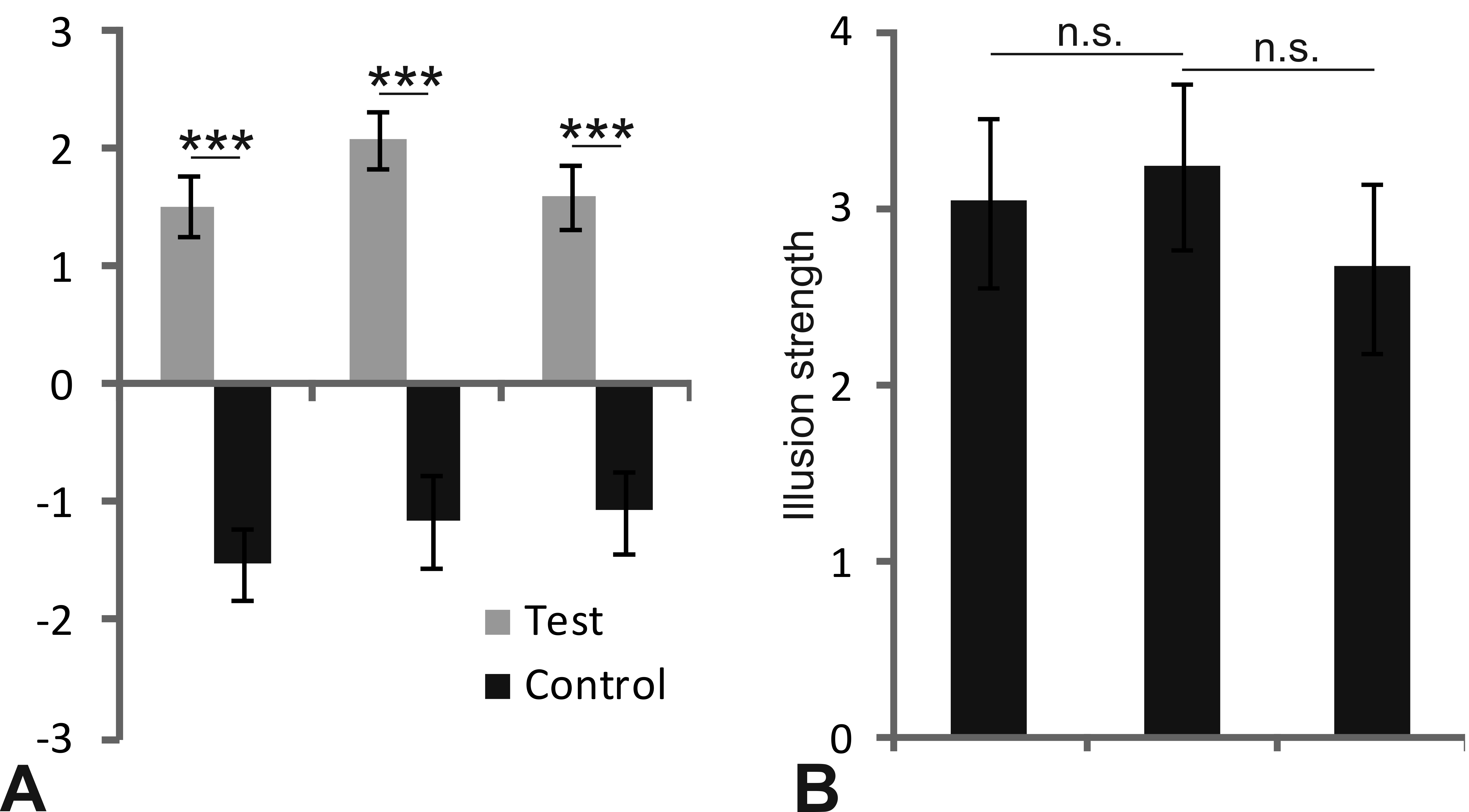

Supplement: Figure S3 — Combined questionnaire results for size perception in Experiments 6 and 7. Results displayed according to statement type (illusion and control) and body size (A), and the illusion strength (defined as average score for illusion statements minus average score on control statement for different body sizes) (B). *** p<0.001, n.s. = difference is not significant. Error bars indicate SEM. (TIF) [file pone.0020195.s003.tif]
