# Supplementary material for: Being Barbie: The Size of One’s Own Body Determines the Perceived Size of the World
Source: PLoS One. 2011 May 25;6(5):e20195. doi: 10.1371/journal.pone.0020195 (PMC3102093; doi:10.1371/journal.pone.0020195)
Supplement: Table S1 — (DOCX) [file pone.0020195.s004.docx]

**Table S1: Questionnaire for experiments 1, 2, and 5**

| During the experiment, there were times when … | |
| --- | --- |
| T1 | … I felt as if the doll's body* was my body. |
| T2 | … it seemed as though the touch I felt was caused by the object touching the doll.* |
| T3 | … it seemed as if I was feeling the touch that was applied to the doll.* |
| C1 | … I felt as if I had two bodies. |
| C2 | … I felt younger** than I actually am. |
| C3 | … I felt as if my body was turning "artificial." |
| C4 | … the doll began to resemble my own body in terms of shape, skin tone, or some other visual feature. |
| S1*** | The hand I saw appeared to me like a hand of a giant. |
| S2*** | The pencil appeared to be gigantic. |

T1–T3: test statements 1–3, C1–C4: control statements 1–4, S1–S2: size statements 1–2.

* The questionnaire for experiment 2 used the term “artificial body” instead of “doll’s body.”

** The questionnaire for experiment 2 used the term “older” instead of “younger.”

*** These questions appeared only in the questionnaire for experiment 5.
