# Supplementary material for: Being Barbie: The Size of One’s Own Body Determines the Perceived Size of the World
Source: PLoS One. 2011 May 25;6(5):e20195. doi: 10.1371/journal.pone.0020195 (PMC3102093; doi:10.1371/journal.pone.0020195)
Supplement: Table S2 — (DOCX) [file pone.0020195.s005.docx]

Table S2: Questionnaire for experiments 6 and 7

| During the first* part of the experiment, there were times when … | |
| --- | --- |
| T1 | … I felt as if the doll's body* was my body. |
| T2 | … it seemed as though the touch I felt was caused by the object touching the doll*. |
| C | … I felt as if I had two bodies. |

T1, T2: test statements 1 and 2, C: control statement.

* The questionnaire was repeated for the second and third artificial bodies.
