# Supplementary material for: Being Barbie: The Size of One’s Own Body Determines the Perceived Size of the World
Source: PLoS One. 2011 May 25;6(5):e20195. doi: 10.1371/journal.pone.0020195 (PMC3102093; doi:10.1371/journal.pone.0020195)
Supplement: Text S1 — (DOCX) [file pone.0020195.s006.docx]

**Text S1**

In experiments 1 and 2, participants rated three test statements and four control statements after synchronous or asynchronous visuotactile stimulation (see Table S1). In both experiments, participants rated each of the test statements significantly higher after synchronous stimulation than asynchronous (small body: t14 > 2.398, p < 0.05; large body: t13 > 3.385, p < 0.01; paired t-tests) (see Figure S1).

At the very end of experiments 6 and 7, the participants were given a questionnaire in which they rated the strength of the illusion (as in experiments 1 and 2). In all body conditions, illusion statements were rated significantly higher than control statements (t26 > 6.945, p < 0.001; paired t-tests) (see Figure S2A). It is noteworthy that the illusions were of similar strengths across the three different body sizes used (defined as mean illusion statement rating minus control statement rating) (t26 < 1.562, p > 0.130; paired t-tests) (see Figure S2B). Thus, consistent with the results of experiments 1–4, the illusion worked equally well for bodies of very different sizes. This precludes the possibility that differences in the vividness of the illusions would have biased the object size reports when the different bodies were used.
